# Visual Attention to Novel Products – Cross-Cultural Insights From Physiological Data

**DOI:** 10.3389/fpsyg.2022.840862

**Published:** 2022-09-08

**Authors:** Isabella Rinklin, Marco Hubert, Monika Koller, Peter Kenning

**Affiliations:** ^1^Department of Corporate Management and Economics, Zeppelin University, Friedrichshafen, Germany; ^2^Department of Management, Aarhus University, Aarhus, Denmark; ^3^Department of Marketing, Vienna University of Economics and Business, Vienna, Austria; ^4^Faculty of Business Administration and Economics, University of Düsseldorf, Düsseldorf, Germany

**Keywords:** visual attention, attractiveness of novel products, mobile eye-tracking, electrodermal activity, cross-cultural comparison, consumer neuroscience

## Abstract

The study aims to investigate visual attention and perceived attractiveness to known versus unknown (novel) products above and beyond self-report applying physiological methods. A cross-cultural exploratory approach allows for comparing results gathered in the United States and China. We collected field data on physiological parameters accompanied by behavioral data. Mobile eye-tracking was employed to capture attention by measuring gaze parameters and electrodermal activity serves as indicator for arousal at an unconscious level. A traditional scale approach measuring perceived attractiveness of known versus novel products provide insights at a conscious level. US-American and Chinese consumers in our sample indeed process novel products differently. This can be observed at an unconscious and conscious level. Different gaze movements and arousal levels are observed at an unconscious level. Regarding behavioral data, the level of vertical orientation shapes the perceived attractiveness of novel products at a conscious level. The study showcases how physiological methods complement behavioral ones when investigating visual attention to products. It underlines varying conscious as well as unconscious visual attention and attractiveness ratings comparing known versus novel products, driven by cultural differences. Data from a field setting further enrich the implications derived for new product development and applied marketing.

## Introduction

Launching novel products often cuts both ways, it either paves the path for further economic growth or it ends in loss and troubles. Hence, for companies engaging in new product development, it is vital to understand which factors contribute to a new product’s success. In a meta-analysis, Evanschitzky et al. (2012) report product-, strategy-, process-, market- place-, or organizational characteristics to have an effect on new product success. Being innovative positively affects specific economic performance values, like firm value (i.e., market to book ratio), market position (i.e., sales, market share), or financial positions (i.e., return on investment) (Rubera and Kirca, 2012). In turn, this effect can be mainly attributed to the success of novel products. However, products are only successful if they are bought by consumers. Hence, it is all about understanding the consumers’ perceptions and attractiveness ratings which serve as key to new product success.

A substantial percentage of newly developed products fail because consumers do not understand them or do not accept them (Feiereisen et al., 2008; Evanschitzky et al., 2012). Especially the decision-making process of consumers to buy novel products is often characterized by a high degree of complexity (Broniarczyk and Alba, 1994; Mukherjee and Hoyer, 2001). Visual attention plays a crucial role in this context. It serves as gatekeeper for further information processing (Hoyer et al., 2018). Proper processing of information is especially vital when it comes to the understanding and acceptance of novel products. Perceived risk and uncertainty further play a crucial role in this context (Mitchell and Boustani, 1993). In turn, cultural aspects shape the levels of perceived risk and associated levels of arousal (Gierlach et al., 2010). The increased speed at which companies develop novel products has led to a rise in studies on visual attention, associated perceived risk and arousal as well as information processing of consumers regarding novel products (Bettman et al., 1998; Mukherjee and Hoyer, 2001; Arts et al., 2011; Kardes and Wyer, 2013; Hubert et al., 2017). Moreover, in today’s globalized world and with regard to strategies in international marketing, the perception and production of new products needs to be discussed in a multicultural setting (Evanschitzky et al., 2012; Rubera and Kirca, 2012). Having a more comprehensive knowledge of how novel products are perceived by consumers in cross-cultural settings is crucial. Previous studies have already shown that cultural differences drive the perception of products and objects in general (e.g., Ishii et al., 2003; Strombach et al., 2014). As the processes of visual attention and perception are often related to complex unconscious automatic processes (Messner and Wänke, 2011) it is difficult to measure these concepts using self-report data only. Physiological as well as gaze parameters allow for a more comprehensive understanding in this regard (Shimojo et al., 2003). Prior intercultural studies (e.g., Bao et al., 2003; Shavitt et al., 2006) did also not account for socioeconomic changes such as Chinese increasingly focusing on Western behavior (Davis, 2013).

Against this background, the present exploratory study focuses on a cross-cultural comparison of visual attention and attractiveness ratings of already known versus novel products. In particular, the study investigates the visual information processes regarding cars that consumers already are familiar with as well as prototypes of cars that are new to them (novel products). With respect to unconscious processes, eye-tracking was applied to measure consumers’ visual attention and electrodermal activity (EDA) measures were included for investigating their arousal levels, which are according to literature related to psychological correlates such as positive/negative emotion, mental effort, task difficulty, risk perception/uncertainty, and anxiety (Holper et al., 2014). The field setting applied in the United States and China allows for a cross-cultural comparison and increases external validity (Kopton and Kenning, 2014; Gneezy, 2017). With regard to the conscious evaluation of the products under scrutiny, a traditional scale approach featuring measures for attractiveness (Brakus et al., 2014) was employed.

The paper contributes to the understanding of cross-cultural differences in visual information processing of products in general and novel products in particular. It, potentially, provides insights into product evaluation in terms of attractiveness ratings at both, conscious and unconscious levels. This approach enables to understand consumer behavior in a cross-cultural setting above and beyond mere survey data. Implications within the context of new product development can be derived on a more comprehensive level, as physiological data of gaze parameters as well as electrodermal activity provide insights into the unconscious drivers of stated product ratings. Adding this kind of data enables to draw a more holistic picture of what is really going on inside the consumers’ in terms of cognitive and affective information processing and therefore it might help to come up with ideas to enhance the customer experience and consumers’ value perceptions.

## Theoretical Frame and Hypotheses

In the following, we provide the conceptual frame for the hypotheses tested along the relevant theoretical concepts investigated in the present study. The study provides insights mainly into the areas of visual attention and perceived attractiveness of novel products. Theoretical underpinnings of how uncertainty and culture relate to those key areas are outlined.

### Visual Attention

For human beings vision has been seen as the most dominant sense (Hutmacher, 2019). When it comes to new product evaluation from a consumers’ perspective, visual attention therefore plays a vital role. For the understanding of visual attention and attentional deployment, different antecedents like (1) surrounding context, (2) salience mapping, (3) inhibition of return, (4) eye movement, or (5) scene understanding and object recognition are crucial (Itti and Koch, 2001). Regarding the design aspects in new product development, prior studies have stated that especially visual attention plays an important role in the way that consumers process novel product designs (Johnston et al., 1990; Folkes and Matta, 2004). From a physiological perspective, gaze parameters serve as an indicator for visual inspection (Shimojo et al., 2003). When it comes to the investigation of visual perception of novel products among consumers from different cultures, it is important to note that prior research has shown different gaze parameters comparing Asian and Western cultures. Consumers from Asian nations tend to screen stimuli for short durations, whereas people from Western nations tend to fix their vision on objects for longer durations (Chua et al., 2005; Goh et al., 2009; Peng-Li et al., 2020). Therefore, the following hypothesis is derived:

H1: Chinese consumers in our sample pay shorter visual attention to products than US-American consumers in our sample.

### The Role of Arousal in Visual Attention and in Different Cultural Settings

The novelty of products can create uncertainty in decision-making and is related to perceived risk (Cox and Rich, 1964; Bauer, 1967; Taylor, 1974; Shoemaker and Shoaf, 1975). Prior findings showed that the facets of perceived risk are context-dependent (Campbell and Goodstein, 2001; Featherman and Pavlou, 2003). Perceived risk and uncertainty are accompanied by higher EDA levels and activation in the anterior cingulate cortex of the brain, indicating a higher level of arousal (Critchley, 2002). Psychophysiological arousal covary with risk-sensitive decision-making processes (Studer and Clark, 2011). Furthermore, with respect to the interaction of risk-aversion and culture, it has been shown that Asians tend to be more risk-averse than US-Americans (Bao et al., 2003; Sorrentino et al., 2013). Therefore, it is hypothesized that:

H2: While observing novel products, Chinese consumers in our sample show higher levels of arousal (indicating a potentially higher level of perceived risk) than US-American consumers in our sample.

Moreover, prior research has shown that risk-averse consumers tend to delay the adoption of new products (Aggarwal et al., 1998) and relate them to losses (Bao et al., 2003). This connotation of experiencing a loss might in turn have a negative impact on the consumers’ evaluation of the new product. Therefore, it is assumed that:

H3: Perceived attractiveness of the novel car is lower for Chinese consumers compared to US-American consumers in our sample.

### The Role of Culture in Visual Attention

The theory of individualism versus collectivism has aimed at explaining cross-cultural differences in consumption (Singelis et al., 1995; Shavitt et al., 2006). Closely related to the theory of individualism/collectivism is the approach of vertical versus horizontal orientation. Vertical orientation emphasizes hierarchy versus horizontal orientation focuses more on equality (Triandis and Gelfand, 1998; Rahman and Luomala, 2020). Early consumer research stated that the Chinese were more vertically oriented and collectivistic than US-Americans (e.g., Bao et al., 2003). However, some studies demonstrate that Chinese society (more specifically, people with high income) are increasingly focusing more on individualistic factors than on collectivistic factors (Yan, 2010). Personal wellbeing is one such individualistic factor (Steele and Lynch, 2013). Recent studies also show acculturation to global consumer culture (Czarnecka et al., 2020) as well as a person’s social value orientation playing a role in this context (Moon et al., 2018). Besides those recent developments, it is assumed that power distance and hierarchy is generally still ranked higher in Asian cultures compared to the US (Li et al., 2014; Goa and Zhang, 2022). The following is hypothesized:

H4a: Chinese and US-American consumers in our sample differ in terms of individualism/collectivism.

H4b: Chinese and US-American consumers in our sample differ in terms of vertical versus horizontal orientation.

More specifically, vertically oriented consumers, such as the Chinese, have a high focus on hierarchy, power distance and structure. These consumers rely less on equality in power/status (higher “power distance”; Hofstede, 1984) and focus more on hierarchical structures. Furthermore, consumers from high power distance cultures show lower impulsive buying tendencies, higher self-control, and more deliberate processing (Zhang and Mittal, 2008; Zhang et al., 2012). The greater degree of deliberate processing and the higher self-control are aligned with higher risk-aversion. The subsequent hypothesis is based on these findings and on the conceptual underpinnings regarding the level of arousal:

H5: The vertical orientation of consumers has a significant effect on their attractiveness ratings of the novel car. This effect is moderated by the level of arousal.

## Methodology

### Experimental Setting

The present physiological field studies were conducted in the United States (Los Angeles) and China (Beijing). The 42 participants (US: *N* = 22, Mage = 46.50, SD = 11.97, 14 males; China: *N* = 20, Mage = 35.95, SD = 9.64, 13 males)^1^ were randomly recruited from a base of the targeted segment of potential premium car consumers (e.g., participants with high income, generally well-educated). Different cars were chosen from five well-known car brands in the premium class–subsequently referred to as brands C1, C2, C3, C4, and P. The car brands were neither US-American nor Chinese. Thus, “country-of-origin” effects (Michaelis et al., 2008) can be excluded. While, previous studies on novel product designs lack the use of real prototypes (e.g., Kreuzbauer and Malter, 2005), one of the cars with the presented study was a “real” prototype (P). The prototype showed substantial design changes (compared to the existing model) and had been developed exclusively by a well-known automobile company and was not yet available on the market. In consequence, the use of a real prototype offers the advantages of increasing the external validity of the experiment and of ensuring that the unknown car is not processed differently by participants as a result of being perceived as unrealistic or even artificial.

**FIGURE 1:**
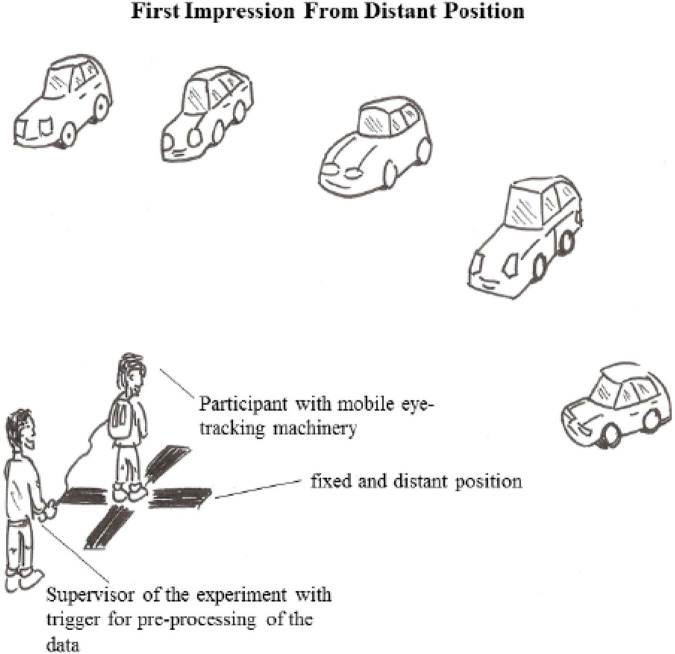
First phase of the experimental setup (own illustration).

**FIGURE 2:**
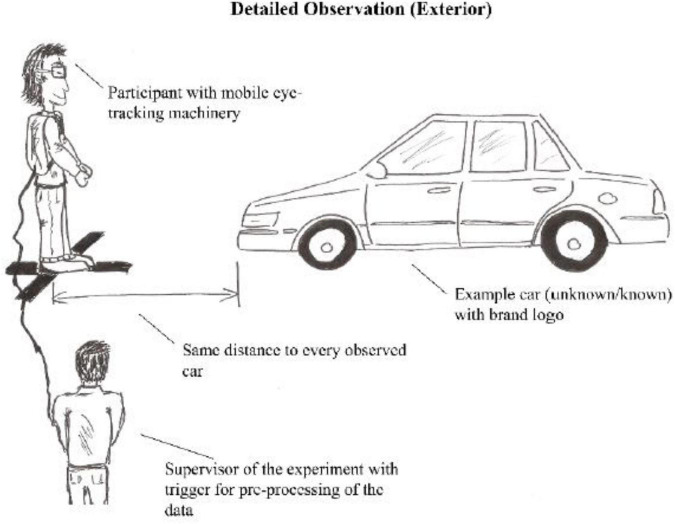
Second phase of the experimental setup (own illustration).

In both cultural groups (China and the United States), a study supervisor led participants through the entire experiment, and for the Chinese group the supervisor was a native speaker of Mandarin. The first sequence of the experiment involved the participants observing all cars from a distant position (capturing their very first impression after opening their eyes from an approximately 5 m distance) (see Figure 1), and the second sequence involved the participants observing each car separately, and from a close-up position (see Figure 2). Each participant was equipped with the mobile neurophysiological technology and ran through the experiment individually. Each participant was instructed by a single experimental supervisor.

### Measurements

For capturing the physiological data a mobile eye-tracking tool (SensoMotoric Instruments, Teltow, Germany) and a mobile EDA tool (edaMove by movisens, with Ag/AgCl electrodes) was used. These tools measure, respectively, visual attention (stationary: Rosbergen et al., 1997; Brasel and Gips, 2008; mobile: Clement et al., 2013; Suher et al., 2014) and arousal (stationary: Kopton et al., 2013; mobile: Kuijpers et al., 2012). They were used during the phases of visual information processing and attractiveness ratings of actual novel cars, in an exhibition hall. For eye-tracking data, the dwell times (ms) were measured associated with visual information processing of each car. The participants looked from a fixed place from which the automobiles were positioned in a way so that the participants could observe the front-side perspective of each car (see Figures 1, 2). In contrast to classical computer lab experiments, the field approach featured different time-durations for each participant’s experimental run. Therefore, each EDA integral (μ) was normalized for each stimulus condition concerning time-duration and was furthermore log-transformed (Bach et al., 2010). As relative values (individual baselines) were used for each participant, it is possible to compare the responses of the Chinese and the Americans without any potential bias due to differences in body temperature or respiration levels.

To determine cultural differences, a traditional rating scales approach was applied. The Horizontal (H)/Vertical (V)–Individualism (I)/Collectivism (C) scale (e.g., Triandis and Gelfand, 1998) (with a nine-point Likert scale range from 1 = “strongly disagree” to 9 = “strongly agree”; HI: α = 0.81; HC: α = 0.89; VI: α = 0.72; and VC: α = 0.76) was used. Attractiveness ratings of the cars served as key behavioral dependent variables. Participants were asked to indicate their general liking for the cars’ exterior designs according to a five-point Likert scale (1 = “extremely attractive” and 5 = “not attractive at all”) similar to Brakus et al. (2014). In order to avoid any language induced bias, scales were translated to Mandarin and American English by a native speaker.

### Results

#### Manipulation Check

Responses from all participants measuring attractiveness were generally high for all cars [Median (Mdn)_C1 = 2; Mdn_C2 = 2; Mdn_C3 = 2; Mdn_P = 1.5] with one exception (Mdn C4 = 3). Friedman’s ANOVA for comparison of all cars was significant, χ2(4) = 37.56, *p* < 0.001. However, Bonferroni-corrected pairwise follow-up tests showed significant differences only for brand C4 (P-C4: *T* = −1.91, adj. *p* < 0.001; C1-C4: *T* = −1.06, adj. *p* = 0.021; C2-C4: *T* = −1.16, adj. *p* = 0.008; C3-C4: *T* = −1.18, adj. *p* = 0.006) (see Table 1). The other comparisons were insignificant. Thus, C4 was excluded from analysis.

**TABLE 1:** Manipulation check: Paired comparisons for Friedman’s ANOVA.

Comparisons	Test statistic	Stand. test statistic	*p*	Adj. *p*
Car P–Car C1	−0.85	−2.45	0.014	0.014
Car P–Car C2	−0.75	−2.17	0.030	0.030
Car P–Car C3	−0.73	−2.11	0.035	0.035
Car P–Car C4	−1.91	−5.52	0.000	<0.001
Car C1–Car C4	−1.06	−3.07	0.002	0.002
Car C2–Car C4	−1.16	−3.35	0.001	0.008
Car C3–Car C4	−1.18	−3.42	0.001	0.006
Car C2–Car C1	0.10	0.28	0.783	1.00
Car C3–Car C1	0.12	0.35	0.730	1.00
Car C3–Car C2	0.02	0.35	0.069	1.00

#### Results of Eye-Tracking Measures

Eye-tracking results from independent *t*-tests (for C2) and from Mann-Whitney tests for non-normally distributed variables (C1, C3, P)^2^ showed that during the first phase of visual processing of the cars, Chinese consumers significantly showed shorter dwell times for all known cars [car C1: Mdn_US = 1499.50, Mdn_China = 166.40, *U* = 85.00, *z* = −2.00, *p* = 0.024; car C2: M_US = 3685.97, SD = 2359.66; M_China = 1622.05, SD = 1538.38, *T*(32) = 2.93, *p* = 0.002; car C3: Mdn_US = 2728.10, Mdn_China = 1131.30; *U* = 88.00, *z* = −1.89, *p* = 0.003] (see Table 2), affirming H1. For the prototype (P), it was found only a marginally significant difference (Mdn_US = 4151.30; Mdn_China = 2446.50, *U* = 96.00, *z* = −1.61, *p* = 0.056).

**TABLE 2:** Statistics of dwell time.

	Americans (*N* = 19)		Chinese (*N* = 15)		Comparisons	
	M (SD)	Mdn	M (SD)	Mdn	Mann–Whitney *U* (z)/*T*-test	*p*
Car C1*	2522.926 (3028.342)	1499.50	1136.25 (1583.53)	166.40	*U* = 85.00 *z* = −2.00	0.024
Car C2	3685.974 (2359.661)	3892.70	1622.05 (1538.38)	1365.80	*T* = 2.927 (*df* = 32)	0.002
Car C3*	3432.000 (2892.018)	2728.10	2236.37 (2538.39)	1131.30	*U* = 88.00 *z* = −1.89	0.003
Car P*	5076.168 (3867.421)	4151.30	3572.25 (4169.86)	2446.50	*U* = 96.00 *z* = −1.61	0.056

*Non-parametric (based on Kolmogorov–Smirnov tests).

#### Results of Electrodermal Activity Measures

For data pre-processing prior to the main EDA analyses, each participant’s overall mean arousal level was computed for all cars (with the mean arousal level for each car’s front, sides, and rear considered).^3^ This allowed to capture the consumers’ complex overall impressions of each car and to account for differences between the front of the car and the other sides of it (see Table 3).

**TABLE 3:** Statistics of arousal levels.

Stimulus	Americans (*N* = 21)		Chinese (*N* = 19)		Comparisons	
	M (SD)	Mdn	M (SD)	Mdn	Mann–Whitney *U* (*z*)/*T*-test	*P*
Car C1	0.2417 (0.169)	0.20	0.33 (0.18)	0.34	*T* = −1.67 (*df* = 38)	0.052
Car C2*	0.21 (0.15)	0.15	0.30 (0.18)	0.30	*U* = 130.00 *z* = −1.88	0.031
Car C3*	0.19 (0.11)	0.15	0.30 (0.18)	0.33	*U* = 118.00 *z* = −2.02	0.022
Car P	0.22 (0.13)	0.18	0.36 (0.21)	0.33	*T* = −2.60 (*df* = 38)	0.007
Car Front C1	0.25 (0.15)	0.15	0.32 (0.19)	0.32	*T* = −1.31 (*df* = 38)	0.099
Car Front C2*	0.25 (0.16)	0.19	0.34 (0.24)	0.32	*U* = 155.00 *z* = −1.21	0.114
Car Front C3**	0.22 (0.13)	0.16	0.36 (0.27)	0.30	*U* = 135.00 *z* = −1.55	0.061
Car Front P*	0.24 (0.16)	0.17	0.42 (0.29)	0.39	*U* = 137.00 *z* = −1.69	0.047

*Non-parametric (based on Kolmogorov–Smirnov tests).

**Highly significant Shapiro–Wilk Test, so that non-parametric test was implemented.

The independent *t*-tests (C1, P) and Mann–Whitney tests (C2, C3) indicated significant differences between American and Chinese participants [car C1: M_US = 0.24, M_China = 0.33, *t*(38) = −1.67, *p* = 0.052; car C2: Mdn_US = 0.15, Mdn_China = 0.29, *U* = 130.00, *z* = −1.88, *p* = 0.031; car C3: Mdn_US = 0.15, Mdn_China = 0.33, *U* = 118.00, *z* = −2.02, *p* = 0.022]. Car P showed the highest significant difference [M_US = 0.22, M_China = 0.36, *t*(38) = −2.60, *p* = 0.007]. Furthermore, only the arousal levels corresponding to the car fronts, each of which is described as the “car’s face” and which characterize the car’s design (Keaveney et al., 2012), are processed further. These results show no significant differences for the known cars. However, they show significant differences for the front of the prototype P (Mdn_US = 0.17, Mdn_China = 0.39, *U* = 137.00, *z* = −1.69, *p* = 0.047), affirming H2.

Regarding H3, focusing on the conscious level of visual information processing *via* asking for the attractiveness ratings, it was found that Chinese participants (Mdn_China = 2.00) indicated significantly lower ratings for the prototype than US-American participants (Mdn_US = 1.00, *U* = 156.00, *z* = −1.76, *p* = 0.039), affirming H3. For the three known cars, the differences between Americans and Chinese were not significant (car C1: Mdn_US = 2, Mdn_China = 2.50, *U* = 185.00, *z* = −0.92, *p* = 0.179; car C2: Mdn_US = 2, Mdn_China = 2, *U* = 193.00, *z* = −0.72, *p* = 0.24; car C3: Mdn_US = 2, Mdn_China = 2, *U* = 206.50, *z* = −0.36, *p* = 0.361).

#### Culture and Arousal as Moderators

Individual cultural differences were captured by using traditional self-report scales for the cultural differences of individualism (vertical/horizontal orientation), collectivism (vertical/horizontal orientation), horizontal orientation (individualism/collectivism), and vertical orientation (individualism/collectivism). There was no significant difference between Chinese and American participants with regard to “individualism” [M_US = 5.97, SD = 1.01, M_China = 6.07, SD = 1.37; *t*(40) = −0.28, *p* = 0.391] and “collectivism” [M_US = 6.97, SD = 1.11, M_China = 6.62, SD = 1.44; *t*(4) = 0.90, *p* = 0.186]. Therefore, H4a had to be rejected. However, results showed differences between the Chinese and American participants concerning horizontal orientation (Mdn_US = 7.56, Mdn_China = 6.94, *U* = 132.50, *z* = −2.21, *p* = 0.014) and vertical orientation [M_US = 5.36, SD = 1.31, M_China = 6.02, SD = 1.34; *t*(40) = −1.60, *p* = 0.059], affirming H4b (see Table 4).

**TABLE 4:** Statistics of cultural differences: Combined factors.

Cultural differences	Americans (*N* = 22)		Chinese (*N* = 20)		Comparisons	
	Means (SD)	Mdn	Means (SD)	Mdn	Mann–Whitney *U* (*z*)/*T*-test	*p*
Individualism (V and H orientation)	5.97 (1.01)	5.94	6.07 (1.37)	5.80	*T* = −0.28	0.391
Collectivism (V and H orientation)	6.97 (1.11)	7.19	6.62 (1.44)	6.72	*T* = 0.90	0.186
Vertical orientation (I and C)	5.36 (1.31)	5.23	6.02 (1.34)	6.00	*T* = −1.60	0.059
Horizontal orientation (I and C)*	7.56 (0.83)	7.56	6.67 (1.46)	6.94	*U* = 132.50 *z* = −2.21	0.014

*Non-parametric (based on Kolmogorov–Smirnov tests).

Finally, to test H5, stating that the individual vertical orientation has an effect on the attractiveness rating of the novel car, a factorial ANOVA (bootstrapping, within-subject design) with attractiveness of P being the dependent variable (*z*-transformed) was computed. Moreover, we tested a potential moderating effect of arousal. The independent variables of vertical orientation (mean of factor sum) and arousal relating to prototype P were binary coded by a median split [Mdn (vertical orientation) = 5.78; Mdn (overall arousal level) = 0.28]. The results demonstrate a marginally significant main effect of consumers’ vertical orientation on the attractiveness ratings for the novel car (prototype P) [*F*(1,15) = 3.09, *p* = 0.099, η_p^2 = 0.171]. The arousal relating to car P showed no main effect. However, the interaction effect turned out to be highly significant [*F*(1,15) = 18.74, *p* = 0.001, η_p^2 = 0.55]. Follow-up tests with confidence intervals based on 1000 bootstrap samples indicated marginally significant differences between consumers with low versus high vertical orientation regarding their attractiveness ratings (M_diff = 0.65, 95% CI [−0.54, 1.22], *p* = 0.081). However, there is no significant difference regarding the arousal of the novel car (prototype P) (M_diff = 0.26, 95% CI [−1.13, 0.86], *p* = 0.251) (see Table 5).

**TABLE 5:** Statistics of bootstrapped factorial ANOVA: Cultural differences and arousal level for novel product.

Dependent variable: Attractiveness rating for novel product *P* (*z*-transformed)				
**Independent variables**	**Sum of squares**	***F***	***p***	$\eta_{\text{p}}^{2}$
Arousal level (P)	0.26	0.49	0.495	0.03
Vertical orientation	1.61	3.09	0.099	0.17
Interaction (Arousal level × Vertical orientation)	9.75	18.74	0.001	0.56
[*R*^2^ = *0.556*.]				

*Levene’s test showed non-significant results.*

## Conclusion, Limitations, and Further Research

Overall, the present small-scale study contributes to a range of areas in consumer research. First, it presents new exploratory findings in the area of cultural differences in consumers’ visual attention of novel products, by providing physiological data from a field study enhancing external validity of the contribution.

Second, the study demonstrates that Chinese and American consumers included in our sample differ in their way, how to visually engage with products. This finding is crucial for any new product development aiming to be launched across different cultures. The cultural differences in visual attention are specifically based on unconscious processes (such as differences in gaze parameters as well as in arousal levels). These findings underline the relevance of investigating cultural differences with physiological measures in addition to behavioral ones.

Third, the analyses reveal that Chinese and US-American consumers included in our sample approach novel products differently. Vertical orientation in particular seems to shape the perceived attractiveness of the novel product. The level of arousal (measured at an implicit level), turns out to be the driving force in this context. Although these findings are already intriguing, further studies would be valuable to test the robustness of the differences found. Further analyses could also focus on a potentially moderating role of perceived uncertainty, thereby strengthening the findings with regard to the differences in arousal levels. Moreover, prior studies have revealed that Chinese participants show lower impulsive buying tendencies due to triggered self-control (Zhang et al., 2012). Because uncertainty could also be managed with higher self-control, it would be useful to observe whether the reaction to uncertainty resulting from novel product changes over time (for instance, after self-control exhaustion for the different cultures). Additionally, the findings provide an indication that Chinese consumers who are targeted for premium products like cars also tend to be more individualistic, like Western consumers. This cultural finding, potentially, demonstrates that socioeconomic change in China is shaping new cultural approaches, which is an area of inquiry that should deserve substantial attention from researchers in the near future. The present study shows that among present-day consumers, US-Americans and Chinese differ predominantly on the basis of divergent horizontal and vertical approaches.

Due to the small sample size, which is often the case in psychophysiological studies (Riedl et al., 2020), the results of the present study are exploratory in nature. Future research should build upon our findings and test whether the effects found also hold for a broader sample.

Moreover, we also suggest including a more diverse set of stimuli as well as further features of the cars tested. Regarding a potential psychological explanation, further research in this context should also account for further socio-demographic variables of the respondents, including individual personality characteristics beyond variables focusing on cultural difference. Doing so would enable to get a more comprehensive picture on different market segments. Focusing not only on evaluations on an attitude-level but also on intentions or actual purchase behavior would further enhance the practical implications of the topic.

Further research could include studies manipulating particular features and element of the cars as well as to include overall evaluations of those features beyond mere visual aspects or where subject asked to mentally compare the cars. This could include qualitative investigations focusing on the mental representations of the features of the cars.

A valuable extension of this research would result from measuring not only the arousal level as such but also including other methods, which are capable of capturing the associated valence component. In this regard, methods like startle reflex modulation (e.g., Koller and Walla, 2012) or fMRI would support the further exploration of the valence changes in EDA levels of consumers with low or high vertical orientations. This would also allow gaining a better understanding of the role of arousal within the context of novel products in a qualitative manner. It would help to develop further insights into the role of positively toned (e.g., excitement or curiosity) or rather negatively toned (e.g., risk and uncertainty) facets within the evaluation and decision-making processes of novel products. Furthermore, moving into the area of brain activation research offers potential for providing information about underlying brain mechanisms that can reveal important insights into the influence that cultural patterns and ongoing cultural changes impose on consumer decision-making. The significance of this line of research extends beyond Chinese and US-American cultures, and beyond the valuable knowledge of responses to novel products. In this way, the present research has potentially opened an avenue of investigation that both needs and deserves an energetic research response.

## Data Availability Statement

The raw data supporting the conclusions of this article will be made available by the first and corresponding author upon request, without undue reservation.

## Ethics Statement

The studies involving human participants were reviewed and approved by IRB, Zeppelin University. The patients/participants provided their written informed consent to participate in this study.

## Author Contributions

IR was the leading author responsible for data collection, data analysis, and the first draft of the manuscript. This manuscript was one part of her Ph.D. thesis “Beiträge zur Integration neurophysiologischer Methoden in das Innovationsmanagement.” MH, MK, and PK contributed equally to the manuscript, revisions, and writing on the provided draft. All authors contributed to the article and approved the submitted version.

## Conflict of Interest

The authors declare that the research was conducted in the absence of any commercial or financial relationships that could be construed as a potential conflict of interest.

## Publisher’s Note

All claims expressed in this article are solely those of the authors and do not necessarily represent those of their affiliated organizations, or those of the publisher, the editors and the reviewers. Any product that may be evaluated in this article, or claim that may be made by its manufacturer, is not guaranteed or endorsed by the publisher.

## Footnotes

1 Age differences between samples are due to the possible pool of participants in the targeted consumer segment. We assume no bias here, because studies on age differences and visual attention are mainly focused on performance and age ranges are much higher between young (around 20 years) and older (around 60 years) participants (see for example Madden et al., 2007).

2 It needs to be mentioned that three US-American and four Chinese participants had to be excluded due to inaccurate eye tracking after re-calibration.

3 It needs to be mentioned that one US-American and one Chinese participants had to be excluded due to inaccurate EDA measurements.
